# A proteomic study of TAR-RNA binding protein (TRBP)-associated factors

**DOI:** 10.1186/2045-3701-1-9

**Published:** 2011-02-25

**Authors:** Ya-Hui Chi, Oliver John Semmes, Kuan-Teh Jeang

**Affiliations:** 1 Institute of Cellular and System Medicine, National Health Research Institutes, Zhunan, Taiwan; 2 Department of Microbiology and Molecular Cell Biology, The Leroy T. Canoles Jr. Cancer Research Center,, Eastern Virginia Medical School, Norfolk, Virginia, USA; 3 Molecular Virology Section, Laboratory of Molecular Microbiology, National Institute of Allergy and Infectious Diseases, National Institutes of Health, Bethesda, MD, USA

## Abstract

**Background:**

The human TAR RNA-binding protein, TRBP, was first identified and cloned based on its high affinity binding to the small hairpin trans-activation responsive (TAR) RNA of HIV-1. TRBP has more recently been found to be a constituent of the RNA-induced silencing complex (RISC) serving as a Dicer co-factor in the processing of the ~70 nucleotide pre-microRNAs(miRNAs) to 21-25 nucleotide mature miRNAs.

**Findings:**

Using co-immunoprecipitation and protein-identification by mass spectrometry, we characterized intracellular proteins that complex with TRBP. These interacting proteins include those that have been described to act in protein synthesis, RNA modifications and processing, DNA transcription, and cell proliferation.

**Conclusions:**

Our findings provide a proteome of factors that may cooperate with TRBP in activities such as miRNA processing and in RNA interference by the RISC complex.

## Findings

MicroRNAs (miRNAs) are important regulators of cellular development, differentiation, proliferation, apoptosis, metabolism, and oncogenesis [1-4]. Cellular miRNAs have also been proposed to function in restricting virus infection of cells [5-11]. The biogenesis of miRNAs in eukaryotic cells has been reviewed extensively elsewhere [12,13]. In brief, RNA polymerase II transcribed primary microRNA transcripts (pri-miRNA) are processed by Drosha in the nucleus to pre-miRNA precursors which are then processed by Dicer in the cytoplasm to mature miRNAs. In analyzing miRNA processing, it was noted that a cellular protein, TAR RNA-binding protein TRBP, binds directly to Dicer [14-17] and is important to the activity of miRNA-mediated RNA-silencing. Moreover, a TRBP-Ago2 composed RNA-induced silencing complex (RISC) has been shown to be needed for optimal miRNA-guided post-transcriptional silencing [18].

TRBP contains two double-stranded RNA binding domains (RBD) [17] and was originally characterized and cloned based on its high affinity binding to the HIV-1 small hairpin leader RNA, TAR [19-21]. Consistent with the notion of miRNA-mediated restriction of viral infection in mammalian cells, loss of TRBP activity through its sequestration by TAR RNA resulted in the enhanced replication of HIV-1 in human cells [22]. To date, TRBP is considered to act inside cells through at least three different mechanisms: it can promote the translation of TAR RNA-containing viral RNAs [23]; it can directly bind and inhibit the interferon (IFN)-induced double-stranded RNA (dsRNA)-activated protein kinase R (PKR) [24]; and as mentioned above, it can be a Dicer co-factor in the miRNA/siRNA RNAi pathway. To gain additional insight into TRBP function, we sought to identify human cellular factors that associate with TRBP. Here, using co-immunoprecipitation (co-IP) and protein-identification by mass spectrometry, we report proteins in a TRBP-proteome.

**Figure 1:**
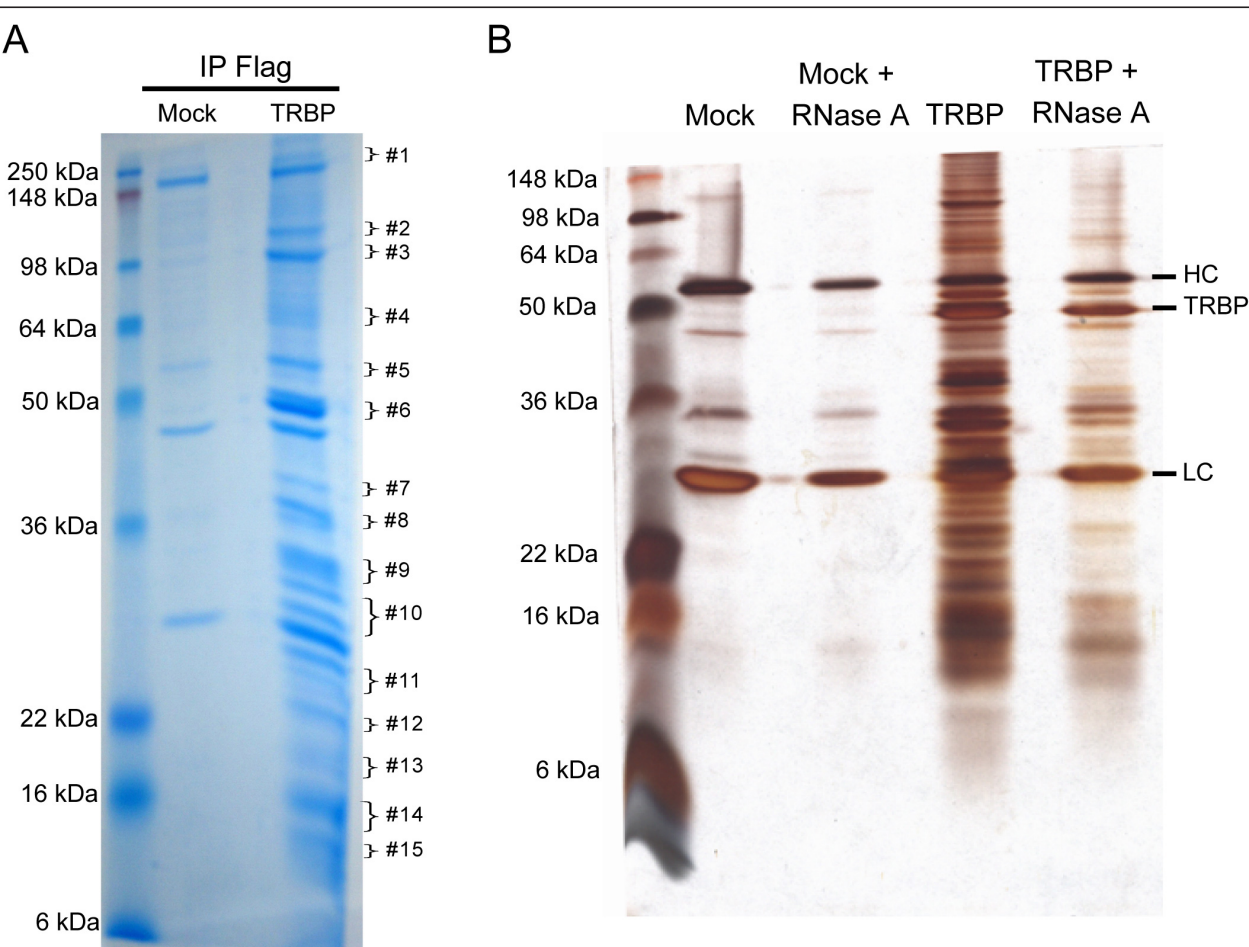
**Gel electrophoresis profile of the TRBP-interacting proteins**. (A) Total cell lysates from HeLa cells overexpressing Flag-TRBP were collected and immunoprecipitated with monoclonal anti-Flag agarose. Flag-TRBP and its interacting proteins were eluted with 3X Flag polypeptides and were subjected to sodium dodecyl-sulfate (SDS) gel electrophoresis followed by colloidal Coomassie staining. Co-immunoprecipitated products using cell lysates with mock transfection were compared. Fifteen gel bands as marked from the mock and TRBP eluents were excised for mass spectrometry analyses. (B) The immunoprecipitated Flag-agarose beads were treated with RNase A for 1 hour and washed three times with RIPA buffer. The remaining binding proteins were eluted with 3X Flag polypeptides and analyzed by gel-electrophoresis and silver staining. Please see Additional file 2, supplementary Materials and Methods, for detailed methodology.

**Figure 2:**
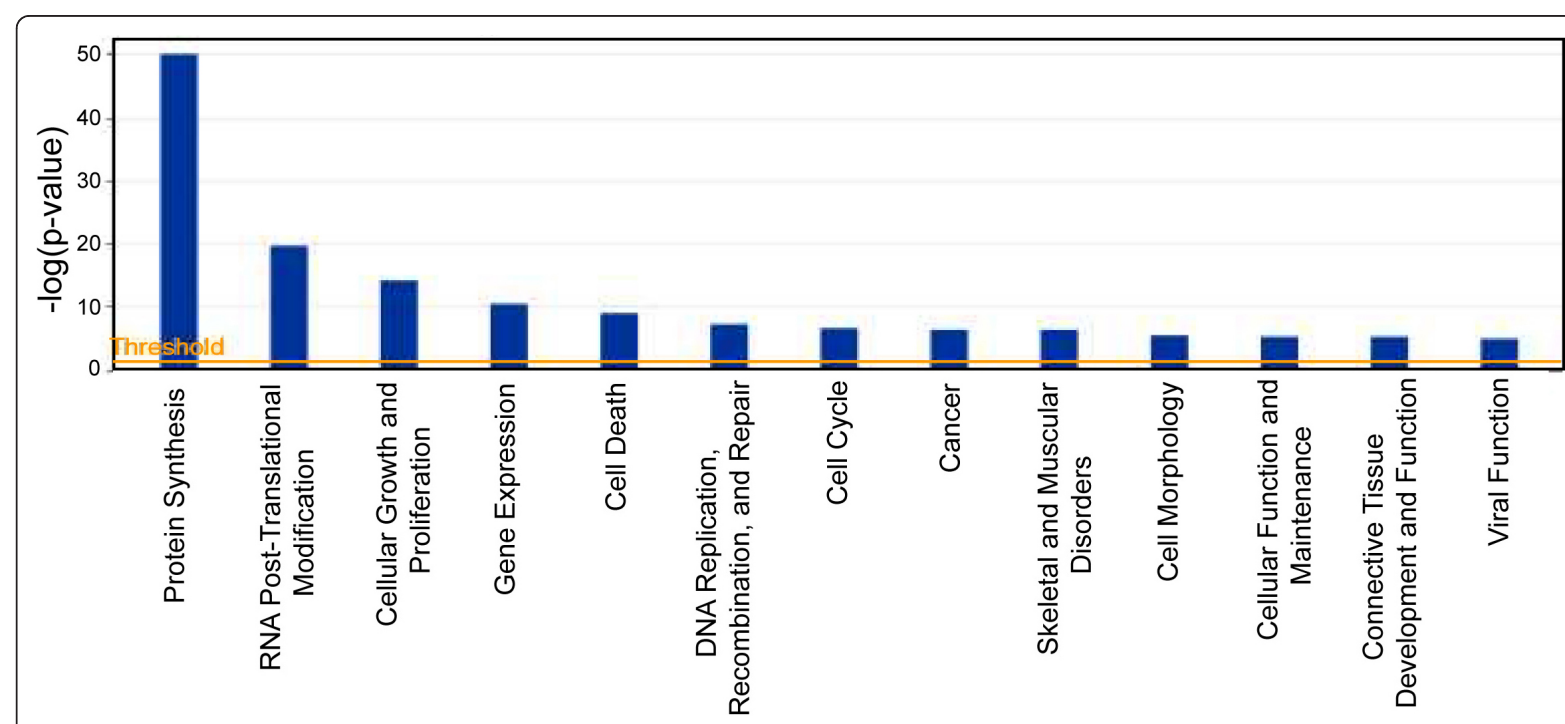
**Functional categorization of the TRBP-interacting proteins**. Categorization of the molecular function of the 160 TRBP interacting proteins was done using the Ingenuity Pathway Analysis software. The y-axis shows statistical relevance of the TRBP-interacting factors in the indicated functional categories. The protein identities of TRBP-associated factors in each functional category are summarized in Table 1.

Figure 1A shows the Coomassie blue stained profile of proteins that co-IP with TRBP. After treating with RNase A, the majority (> 80%) of the co-IP products were reduced in amounts, suggesting that many TRBP-interacting partners in the co-IP are indirectly linked via RNA-association (silver stain, Figure 1B). Next, we compared "Mock" to "TRBP" samples based on silver staining; and we excised the corresponding Coomassie blue stained protein bands that were enhanced in the TRBP sample. These bands were then analyzed by LC-MS/MS spectrometry (Figure 1A). The corresponding gel bands from the "Mock" sample were also excised and analyzed in parallel. By subtracting the "Mock" protein identification data from the "TRBP" protein identification data, 160 "TRBP-associated" factors were identified (Additional file 1, Table S1). The functional relevance (according to the p value computed by the Ingenuity Systems algorithm using right-tailed Fisher Exact Test) of each category of protein factors to established pathways is diagrammed in Figure 2, and the protein identities present in each functional category are listed in Table 1. Similar to a previous report [25], our results show that TRBP is significantly associated with factors in the protein synthesis machinery that is constituted by the 80S ribosome [20 out of 32 proteins (RPSs; ribosomal protein, small subunits) of the 40S ribosomal subunits, and 24 out of 46 proteins (RPLs; ribosomal protein, large subunits) of the 60S ribosomal subunit were identified] [26]. Also identified were several translation elongation factors (*e.g. *ABCF1, EEF1A2, EIF2AK2 and EIF2S1) (Table 1).

**Table 1:** Functional categories of the TRBP-associated complex

Category	Molecules
Protein Synthesis	ABCF1, EEF1A2, EIF2AK2, EIF2S1, PTBP1, RPL3, RPL4, RPL5, RPL6, RPL7, RPL9, RPL11, RPL15, RPL18, RPL21, RPL22, RPL24, RPL30, RPL32, RPL34, RPL35, RPL18A, RPL23A, RPL27A, RPL29 (includes EG:6159), RPL31 (includes EG:6160), RPL7A (includes EG:6130), RPS2, RPS5, RPS6, RPS9, RPS10, RPS11, RPS17 (includes EG:6218), RPS3A
RNA Post-Transcriptional Modification	DDX17, DDX54, EFTUD2, HNRPAB, HNRPC, HNRPUL1, PABPC1, PRPF8, PTBP1, SFRS1, SNRPD1, SNRPD3, SYNCRIP, U2AF1
Cellular Growth and Proliferation	AKAP13, ATP5B, ATP5F1, CCT2, DBN1, DCD, DNAJA3, EIF2AK2, GLTSCR2, HMGA1, HNRPA1, HNRPAB, HNRPC, HNRPF, HNRPM, PHB (includes EG:5245), PPP1R9B, PRPF8, PRPF19, RAP1B, RPS3A, RSL1D1, SART1, SFRS1, TOP1, XRN2
Gene Expression	BASP1, BTF3, CDC5L, CHD4, CSDA, DDX5, DEK, EIF2S1, GTF3C1, GTF3C3, GTF3C4, HMGA1, HNRPAB, IGHMBP2, PARP1, PHB (includes EG:5245), RBM39, RPL6, SOX21, TARBP2, TOP1, TOP2A
Cell Death	DDX5, DIDO1, DNAJA3, EEF1A2, EIF2AK2, EIF2S1, GNL3, HIST1H1C, HMGA1, HNRPA1, HNRPC, HSPA1B, PARP1, PHB (includes EG:5245), PPP1R9B, PRPF19, RBBP4, RPLP0 (includes EG:6175), RPS3A, SLC2A1, TOP1, TOP2A
DNA Replication, Recombination, and Repair	CDC5L, HMGA1, HSPA1B, IGHMBP2, PARP1, PRPF19, TOP1, TOP2A
Cell Cycle	CDC5L, EIF2AK2, GNL3, HMGA1, NOL1, PARP1, PES1, PHB (includes EG:5245), PPP1R9B, PRMT5, RPL5, RPL11, RPL23, TOP2A
Cancer	RPL5, RPL11, RPL23, AKAP13, ASPH, DNAJA3, HMGA1, HSPA1B, PES1, PRMT5, RPS3A
Skeletal and Muscular Disorders	RPL5, RPL11, RPL23
Cell Morphology	AKAP13, ASPH, DNAJA3, HMGA1, HSPA1B, PES1, PRMT5, RPS3A
Cellular Function and Maintenance/Connective Tissue Development and Function	EIF2AK2, EIF2S1
Viral Function	AKAP13, EIF2AK2, HMGA1, PARP1, SFRS1

Although previous reports implicated TRBP as active primarily in the cytoplasm, our mass spectrometry results (Additional file 2. Supplementary Materials and Methods) identified several TRBP-interacting nuclear factors which are known to participate in processes including RNA splicing, cellular growth and proliferation and gene transcription (Figure 2, Table 1; and Additional file 1, Table S1). For example, EFTUD2, HNRPC, PRPF8, PTBP1, SFRS1, SYNCRIP, SNRPD1, SNRPD3 and U2AF1 are components of the small nuclear ribonucleoprotein (snRNPs) that are involved in splicing of pre-mRNAs. Related to gene transcription, TRBP-associated proteins include those found to be active in RNA polymerase II- and III-dependent transcription (Additional file 1, Table S1). Additionally, a TRBP-associated factor, Sox21, is a HMG-box transcription factor that acts in a complex array of repressive and activating transcriptional processes in embryonic stem cells [27]. The transcription co-activator HMGA1 was also identified in our proteomic data (Table 1). Whether TRBP participates in the determination of pluripotency through Sox21 requires further study.

Recently, TRBP truncating mutations were found in human cancers with microsatellite instability [28]. Evidence exists which supports the notion that impaired miRNA processing and destabilization of the DICER1 protein are correlated with tumorigenesis [28]. Because TRBP is known to interact with Dicer and to be involved in mRNA processing, how TRBP and TRBP-interacting proteins contribute to oncogenesis merits future investigation. The current TRBP proteome provides a starting point for initiating those explorations. Our study describes the TRBP-proteome in HeLa cells. Although TRBP was originally identified as the HIV-1 TAR RNA-binding protein, its more recently characterized function in microRNA biogenesis and RISC-activity is widely conserved in all cell types (e.g. epithelial, hematopoietic, mesenchymal cells etc...). Thus, a HeLa-like TRBP-proteome is likely to be found in all types of human cells. We realize that systematic functional validation of the currently identified TRBP proteome will be necessary in order to clarify the biological relevance of each factor. Indeed, some of the TRBP-interacting proteins are being currently characterized.

## Abbreviations

TRBP: TAR-RNA binding protein 2; RISC: RNA-induced silencing complex; TAR: trans-activation responsive element.

## Competing interests

The authors declare that they have no competing interests.

## Authors' contributions

YHC constructed the TRBP expression plasmid, performed co-immunoprecipitations, prepared MS samples, analyzed the results; OJS provided mass spectrometry analyses; KTJ conceived of the study. YHC and KTJ prepared the manuscript. All authors read and approved the final manuscript.

## Supplementary Material

Additional file 1 **Supplementary Table S1**. Pubmed ID, gene name, functional description, hits in proteomics study and alternative symbol for TRBP-interacting factors. Click here for file

Additional file 2 **Supplementary Materials and Methods**. Click here for file
