# Supplementary material for: A proteomic study of TAR-RNA binding protein (TRBP)-associated factors
Source: Cell Biosci. 2011 Feb 25;1:9. doi: 10.1186/2045-3701-1-9 (PMC3125213; doi:10.1186/2045-3701-1-9)
Supplement: Additional file 2 — Supplementary Materials and Methods. [file 2045-3701-1-9-S2.DOCX]

**Supplementary materials and methods**

*Cell culture and transfection.* HeLa cells were maintained in DMEM containing 10% fetal bovine serum (FBS) and supplemented with 2 mM L-glutamine and antibiotics. HeLa cells at 50% confluency in three 15cm culture plates were transfected with a Flag-tagged TRBP (amino acids 22-366) expression plasmid using Lipofectamin (Invitrogen, Carlsbad, CA). 48 hours after transfection, cells were harvested for co-immunoprecipitations.

*Co-immunoprecipitation (co-IP).* The co-immunoprecipitations were performed as described in [23]. In brief, cells were harvested in ice-cold PBS and lysed with RIPA buffer [50 mM HEPES, pH 7.3, 150 mM NaCl, 2 mM EDTA, 20 mM β-gylcerophosphate, 0.1 mM Na_3_VO_4_, 1mM NaF, 0.5 mM DTT and protease inhibitor cocktail (Roche, Indianapolis, IN)] containing 0.5% NP-40. After shearing with a 22 gauge needle, the cleared supernatants were pre-cleaned with protein A agarose for 1 hour at 4^o^C. The cleared lysates were incubated with the monoclonal anti-Flag agarose M2 (Sigma-Aldrich, St. Louis, MO) beads for 16 hours at 4^o^C. After five times of washes with RIPA buffer containing 0.5% NP-40, the co-immunoprecipitated proteins were eluted with 3X Flag polypeptides at 100 μg/mL and analyzed by SDS-PAGE.

*Mass spectrometry analysis*. Protein bands were analyzed by mass spectrometry as described previously [24]. In brief, gel slices were successively treated with 100% acetonitrile, 50 mM DTT and 55 mM iodoacetamide and then dried in a speed-vac. After rehydration and trypsin digestion, peptides were extracted 2 times with 25µl 50% acetonitrile, 5% formic acid and dried in a speed-vac. Peptides were resuspended in 20µl buffer A (5% acetonitrile, 0.1% formic acid, 0.005% heptafluorobutyric acid) and 3-6 µl were loaded onto a C-18 column (The Nest Group, Southboro, MA) and analyzed by a LTQ™ Linear Ion Trap mass spectrometry (ThermoFinnigan, San Jose, CA). Sequence analysis was performed with TurboSEQUEST™ (ThermoFinnigan, San Jose, CA) or MASCOT (Matrix Sciences, London GB) using the non-redundant protein database from National Center for Biotechnology Information (NCBI) web site (<http://www.ncbi.nlm.nih.gov/>).
